# Regenerative medicine in China: demands, capacity, and regulation

**DOI:** 10.1186/s41038-016-0046-8

**Published:** 2016-06-02

**Authors:** Biao Cheng, Shuliang Lu, Xiaobing Fu

**Affiliations:** 1 College of Life Sciences, Medical College of PLA, General Hospital of PLA, Beijing, 100853 People’s Republic of China; 2 The Key Laboratory of Trauma Treatment & Tissue Repair of Tropical Area, PLA, Guangzhou, 510010 People’s Republic of China; 3 Ruijin Hospital, Shanghai Jiao Tong University, Shanghai, 200025 People’s Republic of China; 4 Chinese Academy of Engineering (Division of Medical and Health), College of Life Sciences, Medical College of PLA, General Hospital of PLA, 28 Fu Xian Road, Beijing, 100853 People’s Republic of China

**Keywords:** Regenerative medicine, Stem cell, Tissue engineering, China

## Abstract

Regenerative medicine (RM) is an emerging interdisciplinary field of research. Its clinical application focuses on the repair, replacement, and regeneration of cells, tissues, and organs by approaches including cell reprogramming, stem cell transplantation, tissue engineering, activating factors, and clone treatment. RM has become a hot point of research in China and other countries. China’s main and local governments have attached great importance to RM and given strong support in relevant policies and funding. About 3.5 billion RMB has been invested in this field. Since 1999, China has established about 30 RM centers and cooperates with many advanced countries in RM research and benefits from their cooperation. However, China needs to develop standards, regulations, and management practices suitable for the healthy development of RM. In this review, we focus on its great demand, capacity, and relative regulations.

## Background

Annually, about 100 million Chinese patients will receive treatment by tissue repair and regeneration technologies because of the sharp increase in various injuries, accidents, and diseases of aging. However, the current paradigm of “healing by scar tissue replacement,” regardless of superficial tissues or visceral organs, is stagnating and is far away from the ultimate goal of “regenerating the impaired organ.” Regenerative medicine (RM) is gradually being used to restore the intrinsic repair ability with stem cell transplantation, tissue engineering, activating factors, cell reprogramming, and genetic treatments. RM holds sound promise of restoring organ function that is impaired because of congenital disorders, acquired disease, trauma, and aging by replacing or regenerating cells, tissues, and even organs. RM is expected to transcend traditional organ transplantation and replacement. Stem cell technology and tissue engineering have an outstanding role in RM. RM will become one of the most promising areas of life science in the twenty-first century [1, 2].

In the past 20 years, the RM market has continued to grow in China and other countries such as the USA, Europe, Japan, and Singapore. As the largest developing country, China has impressed the world with its findings in stem cells, tissue engineering, active molecules, and gene therapy as well as its national strategies and regulation of RM. These achievements may benefit China in both disease treatment and society development [3].

## Review

### Strategies, guidance, funding, and industrialization of RM in china

#### National strategies

The central government of China supports the development of RM. In the *2006 National Plan for Long- and Medium-Term Scientific and Technological Development (2006–2020)*, stem cells and RM technologies were the important fields among the five biotechnologies (http://www.gov.cn/gzdt/2009-08/21/content_1398305.htm). Also, local governments have adopted stem cell research as one of the priorities of technological development and provided active support. Relevant government departments and academia have paid close attention to and encouraged the development of RM. In the *Science & Technology on Public Health in China: A Roadmap to 2050*, issued by the Chinese Academy of Sciences (CAS) [4], and the *Study on the Long- and Medium-Term Development Strategy for China Engineering Science and Technology*, issued by the Chinese Academy of Engineering (CAE) (http://news.sciencenet.cn/htmlnews/2012/12/273300-3.shtm), RM was considered a major research field. In the *Roadmap of Translational Medicine in China* issued by CAE, RM and biotherapy are main fields. Industrialization of RM is a part of the “12th Five-Year Planning” and will be nurtured as a source of economic growth.

The strategic science and technology projects from CAS can be divided into “Forward-Looking Strategic Priority Research Program of Science and Technology” and “Construction of Research Centers for Basic and Forefront Scientific Research.” Academia held three Xiangshan Science Conferences on RM, in 2005, 2010, and 2015, to discuss the philosophy, scope, and major breakthroughs needed for the development of RM in China and the key scientific issues to be addressed. In addition, the Xiangshan Science Conference organized seminars on stem cell biology and cloning, strategies for research and development of gene therapy and biomaterials, and tissue engineering.

#### Regulatory and scientific guidance

Policies and regulations reflect that China is gradually strengthening the management of RM research and clinical application. Since 1999, when the Ministry of Health (MH) promulgated the first *Umbilical cord blood stem cell bank management approach (Trial),* about 30 rules and regulations have been issued by the Ministry of Science and Technology (MST), the MH, and the State Food and Drug Agency (SFDA) (Table 1).

**Table 1:** Management specification of stem cell transplantation techniques

Time	Management specification of stem cell transplantation techniques
2006	Unrelated Hematopoietic Stem Cell Transplantation Technique
	Unrelated Hematopoietic Stem Cell Transplantation Collection Technique
2009	Cord Blood Stem Cell Therapy Technology (Trial)
	Tissue-engineering Tissue Transplantation Therapy Technique (Trial)

In 2011, the First National Stem Cell Research Guidance and Coordination Committee was established for the overall design and scientific planning of stem cell research in China. In December 2011, the *Notice on Carrying out Self-inspection and Self-rectification Campaign Regarding Stem Cell Clinical Research and Application* was issued. In 2013, the stem cell clinical research and application rectification lead group of the MH and SFDA formulated the regulations *Management Specification of Stem Cell Clinical Trials (Trial)*, *Management Specification of Stem Cell Clinical Trial Research Base (Trial)*, and *Stem Cell Preparation Quality Control and Pre-clinical Research Guidelines (Trial)*. These regulations will be implemented soon and help in the development of RM in China. In 2015, China MH opens the window to allow the stem cell clinic trial and stem cell research bases which will promote the development of stem cell research and translational application in China.

#### Funding support and resources

**Fig. 1:**
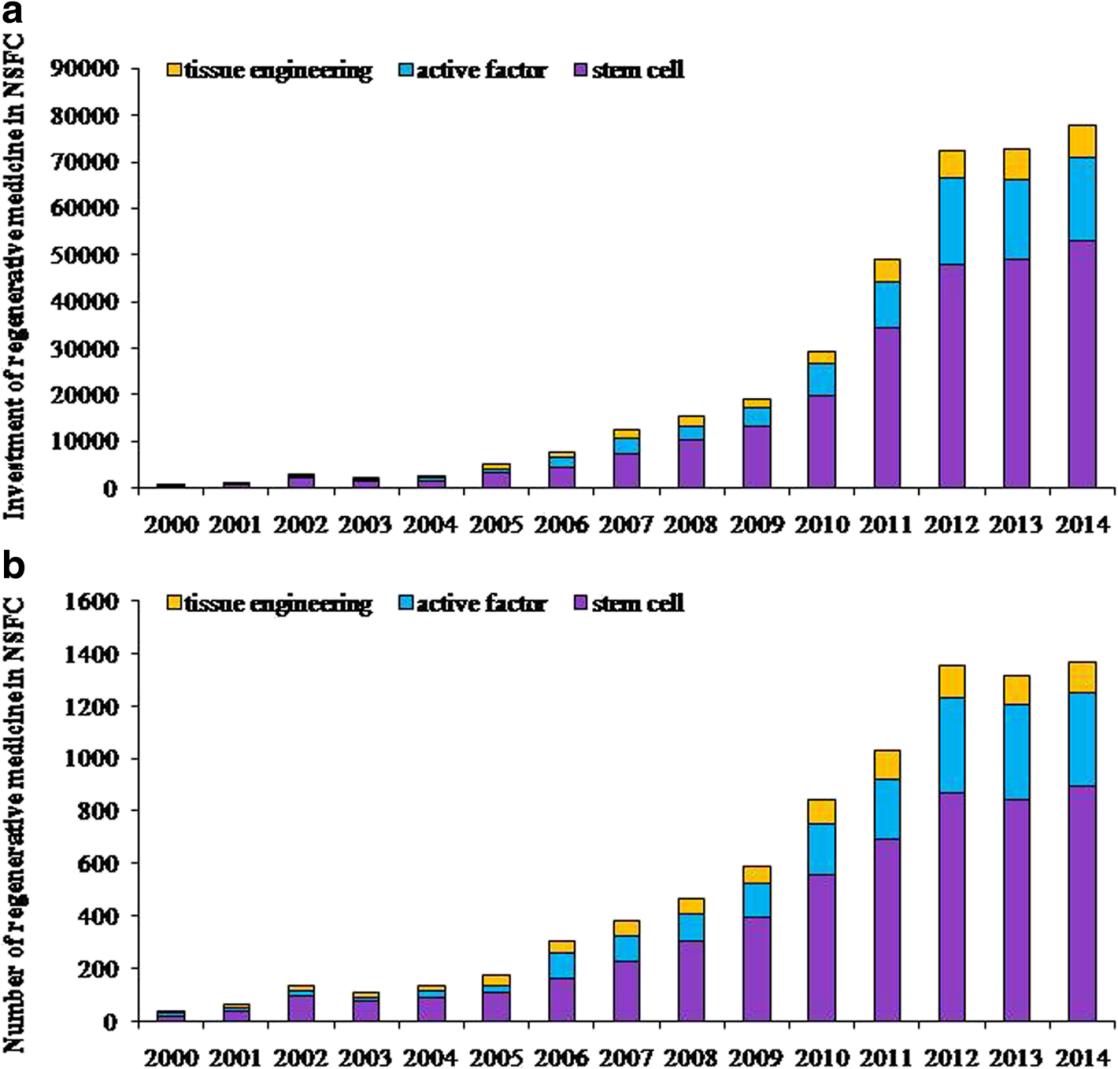
National Natural Science Foundation of China investment in regenerative medicine from 2000 to 2014. **a** Amount of funding. **b** Number of funds

Multiple sources are funding RM studies and translational application. After 1999, the MST approved the National Program on Key Basic Research Project (973 Program) related to tissue engineering, and the stem cell field had the largest number of “973 Program” projects. Research into the clinical transformation and application of stem cell therapy was established in the biotechnology and medical technology field of the “863 Program.” The National Natural Science Foundation (NNSF) funded about 200 million RMB for this study, including 5627 items (Fig. 1a, b). Up to now, about 3 billion RMB from the MST, CAS, and NNSF has been invested in this field. Both the amount of funding and number of projects are increasing annually. Other funding for RM from companies is about 500 million RMB. In 2015, NNSF plans to invest RM research as its “Great Research Plan,” and MST has issued its new scientific research grand with about 21 billion RMB in RM field.

#### Translating outcomes into industrialization

The establishment of centers and technological translational application are important for RM development. Since 1999, China has established about 30 RM centers. These centers are involved in stem cell research and its translational application (e.g., national stem cell east center and national stem cell centers in Tianjin, Qingdao, Wuxi, Taizhou, etc.) (http://www.bioon.com/biology/cell/28500.shtml); national stem cell and RM technology innovation strategic alliance (sponsors and governing members include 27 first-class research institutes, well-known three-A hospitals, several “211 Project” key universities and industry leaders); and a tissue engineering innovation center in Shanghai. In 2011, the first academic workstation for the industrialization of stem cell technology was launched in the Inner Mongolia Autonomous Region. Companies such as Cyagen Biosciences (Guangzhou) and Hangzhou Biowish Technology (Biowish) are specialized in the development and sales of stem cell products. In 2009, NeoStem announced that it had reached an exclusive agreement for strengthening biomedical cooperation with Shanghai enterprises. This agreement aims to establish a network of stem cell collection and treatment centers in Shanghai, Jiangsu, Zhejiang, Fujian, Anhui, and Jiangxi provinces. In 2010, the Beike Stem Cell Bank and Stem Cell Preparation Laboratory successfully passed the ISO 9001 quality management system certification and obtained the qualification certification issued by China Quality Certification Center, becoming the first comprehensive stem cell bank to pass ISO 9001 quality management system certification in China (http://www.bioon.com/industry/enterprisenews/432100.shtml).

### Production in academic fields

**Fig. 2:**
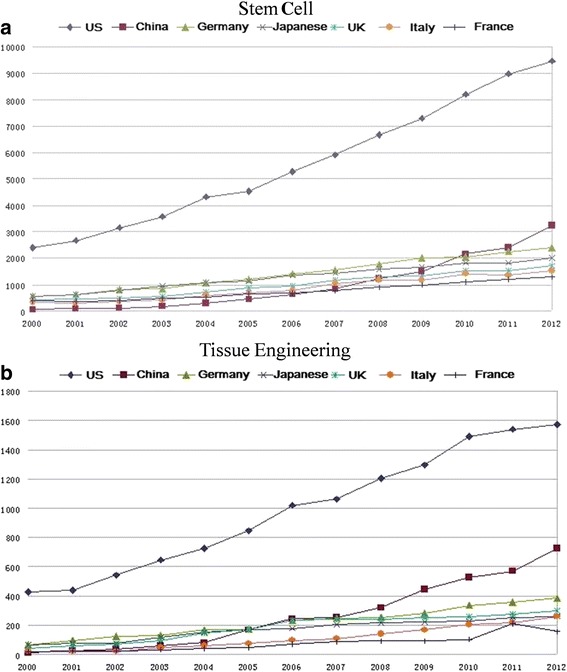
Outputs of regenerative medicine research papers compared with major countries (ISI Web of Knowledge). **a** Stem cells. **b** Tissue engineering

Publication of valued scientific papers is one of the very important indicators to evaluate investment and production in academic fields. The total number of scientific papers dealing with RM has increased quickly in China, as has the number published in leading scientific journals in China and internationally (Fig. 2). Since the 1960s, the USA has published 599 articles about stem cells in *Cell* and its subjournals (36), Germany published 45, Japan 36, and China only 17. Since 2000, the number of annual patent applications for stem cells has increased quickly and amounted to 1333 in 2009. In 2011, the Chinese literature related to stem cells outnumbered that of published by German, Japanese, and UK researchers and ranked second. In 2012, it had increased. In terms of citations, the USA ranked the first, with a mean of 32.2 citations per item. However, citations for Chinese publications are increasing annually, and the mean number is currently 10.19. For patent applications, as of March 2010, the number of China stem cell-related patent applications and patent applications as a patent priority country was ranked the sixth and third in the world, respectively. The USA, Japan, and China have applied for more patents as patent priority countries (http://www.chinainfo.gov.cn/Report/ArticlesView.aspx?aid=7924).

Also, many monographs on stem cells or tissue engineering and RM have been published which play some role in pushing the research of RM in China (Tables 2, 3, and 4).

**Table 2:** Main monographs on regenerative medicine published in China

Time	Name	Author	Publishing company
2008.03	Regenerative Medicine: From Basic to Clinic Research	Fu Xiaobing, Wang Zhengguo, Wu Zuze [5]	Shanghai Scientific and Technical Publishers
2010.05	Regenerative Medicine: Theory and Technology	Pei Xuetao [6]	Science Press
2012.03	Regenerative Medicine	Ding Fei, Liu Wei, Gu Xiaosong [7]	People’s Medical Publishing House
2013.08	Regenerative Medicine: Basic and Clinical Research	Fu Xiaobing, Wang Zhengguo, Wu Zuze [8]	People’s Medical Publishing House
2014.10	Tissue Engineering and Regenerative Medicine	Jin yan [9]	People’s Medical Publishing House
2015.03	Chinese Discipline Development Strategy Regenerative Medicine	Chinese Academy of Sciences [10]	Science Press

**Table 3:** Main monographs on stem cells published in China

Time	Name	Author	Publishing company
1988.01	Basic Hematopoietic Stem Cell Transplant	Wu Zuze [11]	People’s Medical Publishing House
2000.04	Stem Cells and Developmental Biology	Ye Xinsheng, Xu Tian, Tang Xifang, Pei Xuetao [12]	Military Medical Science Press
2000.09	Peripheral Blood Stem Cell Transplantation	Da Wanming, Pei Xuetao [13]	People’s Medical Publishing House
2000.11	Hematopoietic Stem Cells Theory and Transplant Technique	Han Zhongchao [14]	Henan Science and Technology Press
2003.07	Stem Cell Biology	Pei Xuetao [15]	Science Press
2005.03	Stem Cells Theory and Technique	Wang Tinghua, Li Liyan [16]	Science Press
2005.05	Stem Cells Biology	Hu Huozhen [17]	Sichuan University Press
2006.05	Principles, Technology and Clinic of Stem Cell	Zhao Chunhua [18]	Chemical Industry Press
2006.07	Neural Stem Cell Foundation and Application	Zhu Xiaofeng [19]	Science Press
2006.12	Neural Stem Cell	Xu Ruxiang [20]	Military Medical Science Press
2007.03	Hematopoietic Stem Cell Biology and Research Methods	Wang Yaping [21]	Science Press
2007.07	Stem Cell Aging and Disease	Wang Yaping [22]	Science Press
2008.02	Fundamental and Clinic Research of Stem Cells	Yu Yue [23]	Press of University of Science and Technology of China
2010.07	New Technologies of Stem cell Application	Yang Xiaofeng, Zhang Sufen, Guo Zikuan [24]	Military Medical Science Press
2010.01	Clinical Research of Mesenchymal Stem Cells	Wang Tong [25]	People’s Medical Publishing House Co., Ltd
2011.08	The Basis, Ethics and Principles of Clinical Applications of Stem Cells	Jin Kunlin [26]	Science Press
2011.12	Research Legal Regulation of Human Embryonic Stem Cell	Zhao Xu [27]	Shanghai People’s Publishing House
2012.04	Mesenchymal Stem Cells: Basic Research and Clinical Application	Han Zhongchao [28]	Science Press
2012.10	Clinical Research and Application of Stem Cells	Gu Yongquan, Han Zhongcao, Fu Xiaobing [29]	People’s Medical Publishing House
2012.10	Application of Stem Cell Technology for Cardiovascular Diseases	Ma Yitong, Ge Junbo [30]	People’s Medical Publishing House
2012.06	Development Report on Technology and Industry of Stem Cell	Dai Tao, Chi Hui, Fu Xiaobing, Pei Xuetao, Zhou Qi, Li Defu, Lan Baoshi [31]	Science Press
2014.06	Practice of Hematopoietic Stem Cell Transplantation	Huang Xiaojun [32]	People’s Medical Publishing House
2014.07	Clinical Progress of Stem Cell Therapy	Han Zhongchao [33]	Tianjin Science and Technology Translation Publishing Co., Ltd

**Table 4:** Main monographs of tissue engineering published in China

Time	Name	Author	Publishing Company
2002.09	Tissue Engineering	Yang Zhiming [34]	Chemical Industry Press
2003.08	Repair of Medicine and Tissue Engineering	Lao Weide [35]	Chemical Industry Press
2004.06	Principles and Protocol of Tissue Engineering	Jin Yan [36]	Fourth Military Medical University Press
2004.12	The Theory and Practice of Tissue Engineering	Cao Yilin [37]	Shanghai Scientific and Technical Publishers
2005.06	Basic and Clinic Research on Tissue Engineering	Yang Zhiming [38]	Sichuan Scientific and Technological Press
2006.05	Tissue Engineering: A Laboratory Manual	Pei Guoxian, Wei Kuanhai, Jin Dan [39]	Military Medical Science Press
2008.01	Tissue Engineering	Cao Yilin [40]	Science Press
2009.05	Tissue Engineering of Skin	Wu Jinjin, ZhuYoutang [41]	Military Medical Science Press
2010.02	Biomaterials and Tissue Engineering	Xiong Dangsheng [42]	Science Press
2011.03	Stem Cell Tissue Engineering: Basic Theory and Clinical Application	Wang Dianliang [43]	Science Press
2012.01	Introduction to Human Tissue Engineering	Guan Guangju, Jiang Duyin [44]	Shandong University Press
2015.01	Fabrication and Structure Performance of Biodegradable Tissue Engineering Scaffolds	Cui Zhixiang [45]	National Defence Industry Press
2015.06	Medical Polysaccharide Material	Dan Huaping [46]	Science Press

### International collaboration and opportunities for RM in China

Open and cooperative regulations are basic in China. Since 2005, China has cooperated with many countries that are advanced in RM at different levels. Six world-renowned comprehensive RM research institutions from Germany, the USA, Canada, Spain, and The Netherlands established a Regenerative Medicine Coalition (RMC) to jointly promote the research and innovation of RM therapy at cellular levels (Table 5). Even some large foreign pharmaceutical companies, such as General Electric and Sanofi-Aventis, have invested in China for stem cell-related research and achieved relevant results (http://lib.cet.com.cn/paper/szb_con.aspx?id=140472) [47, 48]. Also, the use of theories and skills of RM in military medicine is one of the important fields in the future [49].

**Table 5:** Collaborations between China and other countries in regenerative medicine research

Country of collaboration	Time	Objects
France	2005	Institute of Zoology (IOZ), Chinese Academy of Sciences, and the French National Institute for Agricultural Research
	2007	The Chinese-French Joint Laboratory of Biology of Embryonic Cells of Mammals
Australia	2007	Sino-Australia Center of Excellence for Stem Cell Science
Canada	2007	Monash Immunology and Stem Cell Laboratories (MISCL) was awarded a federal government grant to establish a joint Australia-China Centre for Excellence in Stem Cell Science with Peking University.
	2009	The Ministry of Science and Technology and the Canadian Institutes of Health Research signed a memorandum of understanding.
UK	2005	The Committee of the National Natural Science Foundation of China and the UK Medical Research Council signed a memorandum of cooperation.
	2009	Scottish Centre for Regenerative Medicine (SCRM) and Peking University Stem Cell Research Center (PKUSCRC) established a national international joint research center.
	2012	UK Medical Research Council and the National Natural Science Fund Committee cooperated to jointly fund a stem cell research project.
USA	2009	The California Institute for Regenerative Medicine (CIRM) and the Chinese Ministry of Science and Technology (MST) signed an agreement to collaborate on stem cell research.
Germany	2009	The National Natural Science Fund Committee and the German Science Foundation cooperated to jointly fund a stem cell research project.

## Conclusions

Great demands in RM are not only in China but also in the world. Their theories and key skills or products are used not only in peacetime but also in military field [49]. The Chinese government attaches great importance to this field, and vigorous investments from the government and companies may accelerate the progress in basic research and translational application. Innovation and international cooperation will be emphasized in future studies. Other sound administrative system, laws, technical specifications, and guidelines are very important in pushing their healthy and orderly development.

## References

[1] Nerem RM. Regenerative medicine: the emergence of an industry. J R Soc Interface. 2010;7(Suppl 6):S771–S775. 10.1098/rsif.2010.0348.focus PMC2988277 20843840

[2] Stoltz JF, Isla N, Li YP, Bensoussan D, Zhang L, Huselstein C, et al. Stem Cells and Regenerative Medicine: Myth or Reality of the 21th Century. Stem Cells Int. 2015;2015:734731. 10.1155/2015/734731 PMC4537770 26300923

[3] Fu X. Regenerative medicine research in China: from basic research to clinical practice. Science China Life Sciences. 2014;57(2):155-6. 10.1007/s11427-013-4600-3 24430554

[4] Chen K, Lin Q, Wu J. Science & Technology on Public Health in China: A Roadmap to 2050. Berlin, Heidelberg; Springer Berlin Heidelberg; 2010.

[5] Fu X, Wang Z, Wu Z. Regenerative medicine: from basic to clinic research. Shanghai: Shanghai Scientific & Technical Publishers; 2008.

[6] Pei X. Regenerative medicine: theory and technology. Beijing: Science Press; 2010.

[7] Ding F, Liu W, Gu X. Regenerative medicine. Beijing: People’s Medical Publishing House; 2012.

[8] Fu X, Wang Z, Wu Z. Regenerative medicine: basic and clinical research. Beijing: People’s Medical Publishing House; 2013.

[9] Jin Y. Tissue engineering and regenerative medicine. Beijing: People’s Medical Publishing House; 2014.

[10] Chinese Academy of Sciences. Chinese discipline development strategy regenerative medicine. Beijing: Science Press; 2015.

[11] Wu Z. Basic hematopoietic stem cell transplant. Beijing: People’s Medical Publishing House; 1988.

[12] Ye X, Xu T, Tang X, Pei X. Stem cells and developmental biology. Beijing: Military Medical Science Press; 2000.

[13] Da W, Pei X. Peripheral blood stem cell transplantation. Beijing: People’s Medical Publishing House; 2000.

[14] Han Z. Hematopoietic stem cells theory and transplant technique. Zhengzhou: Henan Science and Technology Press; 2000.

[15] Pei X. Stem cell biology. Beijing: Science Press; 2003.

[16] Wang T, Li L. Stem cells theory and technique. Beijing: Science Press; 2005.

[17] Hu H. Stem cells biology. Chengdu: Sichuan University Press; 2005.

[18] Zhao C. Principles, technology and clinic of stem cell. Beijing: Chemical Industry Press; 2006.

[19] Zhu X. Neural stem cell foundation and application. Beijing: Science Press; 2006.

[20] Xu R. Neural stem cell. Beijing: Military Medical Science Press; 2006.

[21] Wang Y. Hematopoietic stem cell biology and research methods. Beijing: Science Press; 2007.

[22] Wang Y. Stem cell aging and disease. Beijing: Science Press. 2007.

[23] Yu Y. Fundamental and clinic research of stem cells. Hefei: Press of University of Science and Technology of China; 2008.

[24] Yang X, Zhang S, Guo Z. New technologies of stem cell application. Beijing: Military Medical Science Press; 2010.

[25] Wang T. Clinical research of mesenchymal stem cells. Beijing: People’s Medical Publishing House Co.,Ltd; 2010.

[26] Jin K. The basis, ethics and principles of clinical applications of stem cells. Beijing: Science Press; 2011.

[27] Zhao X. Research legal regulation of human embryonic stem cell. Shanghai: Shanghai People's Publishing House; 2011.

[28] Han Z. Mesenchymal stem cells: basic research and clinical application. Beijing: Science Press; 2012.

[29] Gu Y, Han Z, Fu X. Clinical research and application of stem cells. Beijing: People’s Medical Publishing House; 2012.

[30] Ma Y, Ge J. Application of stem cell technology for cardiovascular diseases. Beijing: People’s Medical Publishing House; 2012.

[31] Dai T, Chi H, Fu X, Pei X, Zhou Q, Li D, Lan B. Development report on technology and industry of stem cell. Beijing: Science Press; 2012.

[32] Huang X. Practice of hematopoietic stem cell transplantation. Beijing: People’s Medical Publishing House; 2014.

[33] Han Z. Clinical progress of stem cell therapy. Tianjin: Tianjin Science and Technology Translation Publishing Co., Ltd; 2014.

[34] Yang Z. Tissue engineering. Beijing: Chemical Industry Press; 2002.

[35] Lao W. Repair of medicine and tissue engineering. Beijing: Chemical Industry Press; 2003.

[36] Jin Y. Principles and protocol of tissue engineering. Xi’an: Fourth Military Medical University Press; 2004.

[37] Cao Y. The theory and practice of tissue engineering. Shanghai: Shanghai Scientific & Technical Publishers; 2004.

[38] Yang Z. Basic and clinic research on tissue engineering. Chengdu: Sichuan Scientific and Technological Press; 2005.

[39] Pei G, Wei K, Jin D. Tissue engineering: a laboratory manual. Beijing: Military Medical Science Press; 2006.

[40] Cao Y. Tissue engineering. Beijing: Science Press; 2008.

[41] Wu J, Zhu Y. Tissue engineering of skin. Beijing: Military Medical Science Press; 2009.

[42] Xiong D. Biomaterials and tissue engineering. Beijing: Science Press; 2010.

[43] Wang D. Stem cell tissue engineering: basic theory and clinical application. Beijing: Science Press; 2011.

[44] Guan G, Jiang D. Introduction to human tissue engineering. Jinan: Shandong University Press; 2012.

[45] Cui Z. Fabrication and structure performance of biodegradable tissue engineering scaffolds. Beijing: National Defence Industry Press; 2015.

[46] Dan H. Medical polysaccharide material. Beijing: Science Press; 2015.

[47] Cao N, Liu Z, Chen Z, Wang J, Chen T, Zhao X, et al. Ascorbic acid enhances the cardiac differentiation of induced pluripotent stem cells through promoting the proliferation of cardiac progenitor cells. Cell Res. 2012;22(1):219–36. 10.1038/cr.2011.195 PMC3351910 22143566

[48] Fu X. Regenerative medicine research in China: from basic research to clinical practice. Science China Life Sciences. 2014; 57(2):155-6. 10.1007/s11427-013-4600-3 24430554

[49] Fu X. Military medicine in china: old topic, new concept. Mil Med Res. 2014;1:2–5. 10.1186/2054-9369-1-2 PMC4336117 25722861

